# Identification of NURR1 (Exon 4) and FOXA1 (Exon 3) Haplotypes Associated with mRNA Expression Levels in Peripheral Blood Lymphocytes of Parkinson's Patients in Small Indian Population

**DOI:** 10.1155/2017/6025358

**Published:** 2017-01-31

**Authors:** Jayakrishna Tippabathani, Jayshree Nellore, Vaishnavie Radhakrishnan, Somashree Banik, Sonia Kapoor

**Affiliations:** Department of Biotechnology, Sathyabama University, Chennai 600119, India

## Abstract

Here, we study the expression of NURR1 and FOXA1 mRNA in peripheral blood lymphocytes and its haplotypes in coding region in a small Chennai population of India. Thirty cases of Parkinson's patients (PD) with anti-PD medications (20 males aged 65.85 ± 1.19 and 10 females aged 65.7 ± 1.202) and 30 age matched healthy people (20 males aged 68.45 ± 1.282 and 10 females aged 65.8 ± 1.133) were included. The expression of NURR1 and FOXA1 in PBL was detected by Q-PCR and haplotypes were identified by PCR-SSCP. In the 30 PD cases examined, NURR1 and FOXA1 expression was significantly reduced in both male and female PD patients. However, NURR1 (57.631% reduced in males; 28.93% in females) and FOXA1 (64.42% in males; 55.76% in females) mRNA expression did differ greatly between male and female PD patients. Polymorphisms were identified at exon 4 of the NURR1 and at exon 3 of the FOXA1, respectively, in both male and female patients. A near significant difference in SSCP patterns between genders of control and PD population was analyzed suggesting that further investigations of more patients, more molecular markers, and coding regions should be performed. Such studies could potentially reveal peripheral molecular marker of early PD and different significance to the respective genders.

## 1. Introduction

Parkinson's disease (PD) is the second most common neurodegenerative disorder. PD is characterized by the loss of dopaminergic neurons in the substantia nigra pars compacta which leads to rigidity, bradykinesia, tremor, and postural instability. These symptoms do not develop until about 50–60% of the nigral neurons are lost and about 80–85% of the dopamine content of the striatum is depleted. The exact pathogenic mechanisms underlying the selective dopaminergic cell loss in PD are still not understood, although research points that the disease is caused by a combination of genetic and environmental factors. The prevalence and incidence of PD increase exponentially with age (~1-2% of the population above age 65 and 4–5% above age 85) and are slightly higher in men than in women. Recently in Deccan Chronicle, it has been published that in India the incidence is bound to double in ten years [1]. Magnetic resonance imaging (MRI) and computed tomography (CT) scan are usually unremarkable or may show age specific changes in Parkinson's disease. There is no lab test for PD, so it can be difficult to diagnose. Doctors use medical history and a neurological examination to diagnose it, but 70% of nigral neurons are lost when symptoms appear [2]. Late diagnosis hampers clinical development of new disease-modifying therapies; only alleviating symptoms is possible at present. For this reason, great interest in developing peripheral biomarker for PD has increased.

Molecular biomarkers in body fluids are likely to meet the expectation of unprecedented specificity together with costs that are lower than those of imaging/functional biomarkers. Quantitative assays of cerebrospinal fluid proteomics, serum or plasma metabolomics, and gene expression profile in peripheral blood lymphocytes (PBL) are done in an attempt to identify potential peripheral biomarkers of the disease. Several studies have used PBL to quantity specific changes in dopamine (DA) content, tyrosine hydroxylase activity, DA receptors, and DA transporters in patients with PD [3, 4]. In addition, a significant decrease in mitochondrial complex I activity and a significant increase in caspase-3 activity have been reported in PBL of PD patients [5]. Likewise, genome-wide expression in PBL of PD patients and healthy controls identified co-chaperone protein ST13 as a potential molecular marker of early PD [6]. Based on these data, it has been proposed that PBL can be used for detecting the possible indicators of pathological mechanisms occurring in the brains of patients with PD.

Several studies demonstrated that transcription factors (TFs) like FOXA1/FOXA2, NURR1, PITX3, OTX2, LMX1a/b, and EN1/2 play a key role in mediating mesodiencephalic dopaminergic (mDA) neuron development and remain expressed in adulthood though dopaminergic neuronal maturation is completed [7]. Mutations or variations in genes coding for transcription factors involved in regulation of neuronal development and maintenance of nigrostriatal system function may be risk factors for PD [8, 9]. The human Nurr1 gene has been mapped on chromosome 2q22-23 and is composed of eight exons [10]. It is the key regulator for development of DA neurons and is also considered as a crucial regulator for the expression of several genes involved in PD pathology including DA transporter (DAT), tyrosine hydroxylase (TH), and vesicular monoamine transporter (VMAT2). In addition, variants in NURR1 gene have been found in association with sporadic and familial PD [11–14]. Also, deletion of FOXA1 and FOXA2 in embryonic DA neurons affects the binding of NURR1 to the promoter region of TH and aromatic L-amino acid decarboxylase (AADC) genes leading to a significant loss of TH and AADC expression in the SNpc of embryos and adult mice [15]. The deregulation of FOXA1/2 may also contribute to demise of DA neurons during PD progression in humans [16]. These observations suggest that Nurr1 and FOXA1 are potential candidate genes for PD susceptibility. However, there is no evidence on the genetic variations in FOXA1 gene in PD patients. Although we are diagnosing more patients with PD today, much needs to be done in the Indian context. Hence, the current study has been proposed to explore the peripheral NURR1 and FOXA1 gene expression in PD and to initially look for variation in exon 4 of NURR1 and exon 3 of FOXA1, respectively.

## 2. Subjects and Methods

### 2.1. Subjects

In the present study a total of 60 PBL samples, 20 male PD patients aged 65.85 ± 1.19, 10 female PD patients aged 65.7 ± 1.202, and 30 healthy controls (HC) matched by gender, age, and origin, were collected from Malar hospital, Chennai. The inclusion criteria for PD patients are as follows: being diagnosis with idiopathic PD, patients who are “on” medication (both male and female *N* = 10) and who are medically stable, Minimal Mini Mental State Examination 3 score of 24 or higher, and being able to walk independently indoors without an aid and outdoors with an aid. Control subjects were patient's spouses and volunteers who are free of neurological and psychiatric illnesses. All the female subjects were considered postmenopausal who self-reported the completion of menopause. All subjects or their legally authorized caregivers signed a written consent, approved by the Institutional Human Ethics Committee, Sathyabama University.

### 2.2. RNA Extraction and cDNA Synthesis

Whole blood (1 mL) was anticoagulated with ethylene diamine tetra acetic acid (EDTA), mixed with phosphate-buffered saline (PBS) 1 : 1, layered over HiSep LSM medium (HiMedia), and centrifuged at 400 ×g for 30 min according to the manufacturer's instructions. Peripheral blood mononuclear cells (PBMCs) were isolated and washed in ice-cold PBS for RNA extraction. Total RNA was isolated from PBMCs using the TRIzol Reagent method (Invitrogen) and then cDNA synthesis was performed according to the manufacturer's protocol (Applied Biosystem).

### 2.3. Q-PCR Assay of NURR1 and FOXA1 Gene Expression against Internal Control *β*-Actin

The detailed information on the NURR1, FOXA1, and *β*-actin primers is shown in Table 1. The experiment of QPCR was performed in duplicate with Applied Biosystem power SYBR green following protocol as described: initial denaturation for 5 min at 95°C, followed by 40 cycles of denaturation for 30 sec at 95°C, annealing for 45 sec at 61.5°C, and extension for 45 min at 72°C. *β*-Actin was used as a house keeping gene to standardize the results. The relative quantification of gene expression among the different groups was determined by the formation of 2^−ΔΔCt^ [17].

### 2.4. Detection of Haplotypes by Polymerase Chain Reaction and Single Stranded Conformation Polymorphism (PCR SSCP) Analysis

#### 2.4.1. DNA Isolation and Amplification of DNA

Genomic DNA (200–300 ng) was isolated from each blood sample following the standard protocol [18]. 50 ng of this DNA was added to each 50 *μ*L of polymerase chain reaction (PCR) mix which contained Amplicon master mix and 10 pmol of each primer. The detailed information of the primers used to explore haplotype variation in the exon 4 of NURR1 and exon 3 of FOXA1 are shown in Table 2. As the respective exons are too long for PCR-SSCP analysis it is divided into two reactions 1 and 2, respectively. PCR consisted of initial denaturation at 94°C for 5 minutes, 35 cycles of denaturation at 94°C for 45 seconds, annealing at 60.4°C for 45 seconds, and extension at 72°C for 45 seconds with final extension at 72°C for 10 minutes.

#### 2.4.2. Single Strand Conformation Polymorphism Analysis (SSCP)

Single strand conformation polymorphism (SSCP) is a reproducible, rapid, and quite simple method for the detection of deletions/insertions/rearrangements in the gene, which has been performed as described by Orita et al. [19]. Samples of purified double–stranded PCR product were added to formamide dye (95% formamide, 0.025% xylenecyanol, 0.025% bromophenol blue, and 0.5 M EDTA), denatured at 95°C for 10 minutes, and immediately snap cooled on ice for 15 minutes. Aliquots were loaded on 12% polyacrylamide gel and subjected to electrophoresis for 10 hours at 200 V, 4°C. The gel was stained with 0.1% silver nitrate to visualize the reannealed single stranded PCR products.

### 2.5. Statistical Analysis

Quantitative data were expressed as SEM (standard error mean). The chi-square test or Student's* t*-test was used to test for differences between the PD patients and control subjects in the distribution of gender and age. Multivariate analysis was used to evaluate the differences in the mean value of the NURR1 and FOXA1 mRNA gene expression in PD Patients compared with controls.

## 3. Results and Discussion

This study aimed to detect differences in the expression of genes possibly related to the differentiation, survival, connectivity, and migration of dopamine neurons in peripheral blood of both male and female PD and respective control subjects.

### 3.1. Research Participants

We enrolled 30 consecutive PD patients who were ethnic Indians, diagnosed by the clinical assessments and stringent diagnosis criteria for PD [20] and 30 age matched healthy and neurodegenerative disease control individuals (Table 3) from Fortis Malar Hospital, Adyar. Amongst the PD patients, 20 were men and 10 were women. Further age matched, 30 healthy control individuals were enrolled where 20 were men and 10 were women. The age ranged from 58 to 76 with a mean of 65.85 ± 1.19 years for male PD patients and male controls 68.45 ± 1.282, while in the case of females the age ranged from 59 to 74 years with a mean of 65.7 ± 1.202 for PD patients and female controls 65.8 ± 1.133. From Table 3, there is good evidence that the incidence of PD is more in male population when compared to women. Our finding is in concurrence with the previous reports where men in general are about 1.5 times more likely to develop PD than women, that is, in the ratio of 3 : 1 (male : female) [21].

### 3.2. NURR1 and FOXA1 Expression on PBL of All Study Groups

**Figure 1:**
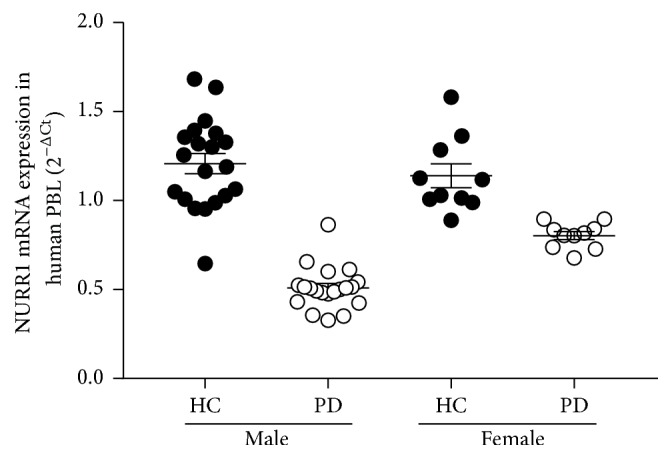
Scatter plot showing NURR1 mRNA expression on PBL in different study groups. Fluorescent reading from Q-PCR was quantitatively analyzed by determining the difference of Ct between Ct of NURR1 and *β*-actin. mRNA expression was determined by using the formation of 2^−ΔCt^. Bars represent mean ± SME measured from duplication assay in blind fashion. *p* < 0.05.

NURR1 is highly expressed in midbrain DAergic neurons [22] as well as other tissues, including PBL [23]. Emerging evidence indicates that impaired NURR1 function might contribute to the pathogenesis of PD: NURR1 and its transcriptional targets are downregulated in midbrain DA neurons that express high levels of the disease-causing protein *α*-synuclein. Recent studies indicated decreased expression in PBL in PD patients independent of medication, disease duration, or severity; NURR1 could be a useful biomarker for PD and related disorders [24, 25]. In our study, we observed reduction in mean mRNA expression of NURR1 in male patients with PD by 57.91% than healthy male controls (*p* < 0.05). To further determine the association of gender with the changes of NURR1 expression, we performed analyses in female PD and found that the NURR1 gene expression was reduced by 28.93% than healthy female controls (*p* < 0.05; Figure 1; Table 3). This data indicates that the decreased expression in NURR1 gene in PD was more robust in male patients and the difference between male PD and female PD was statistically significant (*p* < 0.05; Table 3). Moreover, NURR1 gene expression was more significantly decreased in older and female patients [25]. Focusing on the NURR1 levels in control samples, females showed lesser levels of NURR1 mRNA expression compared to men (*p* < 0.05; Table 3). Pérez-Sieira et al. demonstrated that estrogens are implicated in the upregulation of NURR1 in female rats [26]. Matsuki et al. confirmed that the levels of NURR1 in blood samples from healthy women are associated with the levels of sex hormones, estradiol [E2], and progesterone [27]. As shown in Table 3, the control females in our current study might have lower sex hormones as they are in their postmenopausal phase which might have downregulated the NURR1 expression, a risk factor for PD.

**Figure 2:**
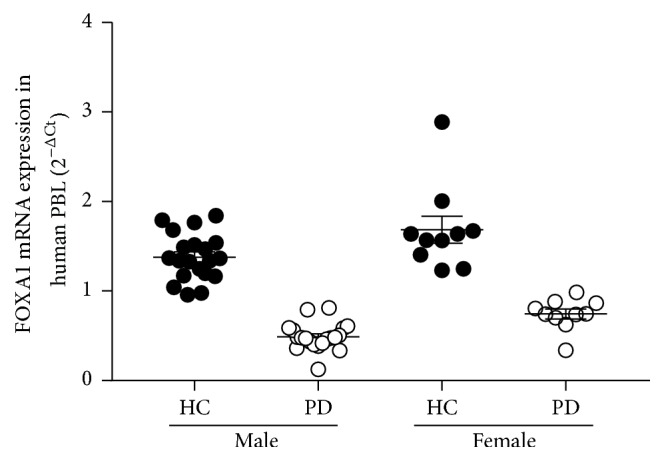
Scatter plot showing FOXA1 mRNA expression on PBL in different study groups. Fluorescent reading from Q-PCR was quantitatively analyzed by determining the difference of Ct between Ct of FOXA 1 and *β*-actin. mRNA expression was determined by using the formation of 2^−ΔCt^. Bars represent mean ± SME measured from duplication assay in blind fashion. *p* < 0.05.

Several datasets from PD patients and PD models showed the downregulation of FOXA1 and FOXA2 expression in the SNpc of PD patients [28–32]. In the current study, the mean mRNA expression of FOXA1 in patients' peripheral blood lymphocytes was analysed and results demonstrated diminished levels in male patients with PD by 64.46% than healthy male controls (*p* < 0.05). Moreover, in female patients it was reduced by 55.74% than healthy female controls (*p* < 0.05; Figure 2; Table 4). This finding suggests that the decreased expression in FOXA1 gene in PD was more robust in male patients and the difference between male PD and female PD was statistically significant (*p* < 0.05; Table 4).

Altogether, these data establishes that lower levels of NURR1 and FOXA1 gene expression were significantly associated with the increased risk for PD in male and female subjects, respectively. Furthermore, our results provide useful information that FOXA1 gene is reduced in PBL of Indian PD patients, indicating its possible systemic involvement in PD.

### 3.3. SSCP Polymorphisms

**Figure 3:**
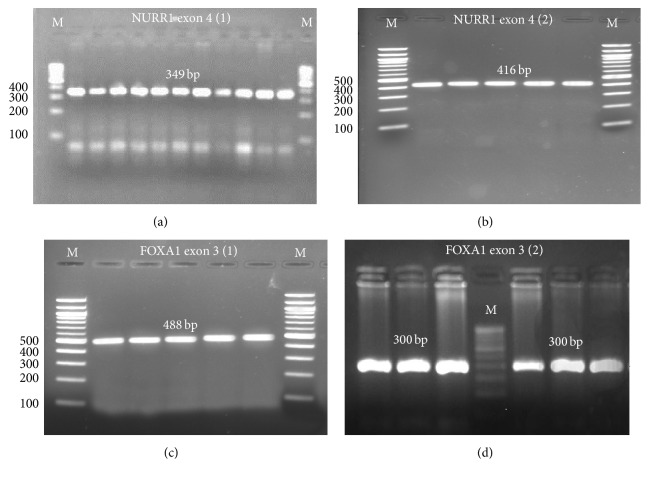
PCR amplification for exon 4 of the NURR1 gene (a-b) and exon 3 of the FOXA1 gene (c-d). The exons were divided into two reactions: 1 and 2, respectively.

It is postulated that variants in NURR1 gene cause decrease in NURR1 mRNA and affect the transcription of gene that encodes TH [9] that could cause dysfunction of dopaminergic neurons and lead to PD [33]. Various studies have reported coding missense mutation in exon 3 of NURR1 (709C>G and 711 C/A), −291Tdel and −245T→G sequence variation in the noncoding exon-1 within the 5′ untranslated region, and mutations in exon 2 at 388 G/A, 35 A/G, and 21 C/G respectively, including some intron regions which markedly attenuates NURR1-induced transcriptional activation, leading to decreased expression being identified in a patient with PD [14, 34]. However, there is no evidence of the genetic variations in FOXA1 gene in PD patients. Hence in the current study, the amplified NURR1 and FOXA1 PCR products in both males and females (Figures 3(a) and 3(b)) were subjected to single-strand conformation polymorphism (SSCP) analysis to clarify whether the variants are disease causing mutations. The exon 4 of the NURR1 gene and the exon 3 of the FOXA1 gene were chosen for the SSCP analysis in two separate reactions, respectively, and the results are described below.

### 3.4. Exon 4 of NURR1

**Figure 4:**
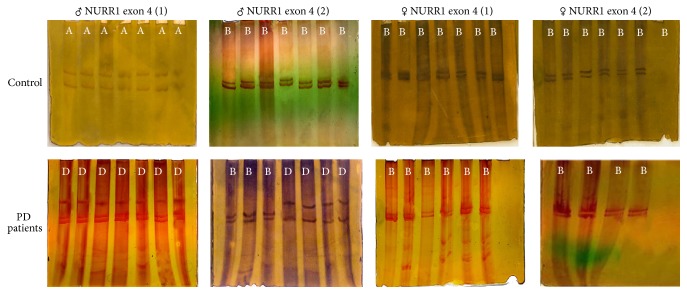
Representative SSCP analyses for exon 4 of the NURR1 gene in male and female PD patients. The exon was divided into two reactions: 1 and 2. A, B, and D represent conformational patterns of the bands found.

Two band patterns were identified for exon 4 of NURR1 gene in male PD controls: patterns A and B (Figure 4). Seventeen out of twenty (85%) male PD patients exhibited migration pattern D (pattern suggesting alterations) with respect to the two reactions of exon 4 of NURR1 gene. Three out of twenty (15%) male PD patients exhibited migration pattern D (pattern suggesting alterations) with respect to reaction 1 and migration pattern B (similar to control) with respect to reaction 2 of exon 4 of NURR1 gene.

The entire female PD controls revealed pattern B with respect to the two reactions of exon 4 of NURR1 gene, which suggested alteration in the pattern of migration, compared to male controls (Figure 4). Interestingly, all the twenty female PD patients exhibited migration pattern B (similar to controls) with respect to the two reactions of the exon 4 of NURR1 gene.

### 3.5. Exon 3 of FOXA1

**Figure 5:**
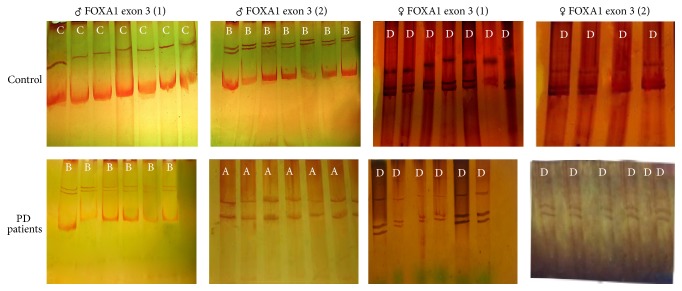
Representative SSCP analyses for exon 3 of the FOXA1 gene in male and female PD patients. The exon was divided into two reactions: 1 and 2. A, B, C, and D represents conformational patterns of the bands found.

Two band patterns were identified for exon 3 of FOXA1 gene in male PD controls: pattern C with respect to reaction 1 and B with respect to reaction 2 (Figure 5). The entire (100%) male PD patients exhibited migration pattern B (pattern suggesting alterations) with respect to reaction 1 and pattern A (pattern suggesting alterations) with respect to reaction 2 of the exon 3 of FOXA1 gene.

A single band pattern was revealed for the entire female PD controls: pattern D with respect to the two reactions of exon 3 of FOXA1 gene, which suggested alteration in the pattern of migration, compared to male controls (Figure 5). Interestingly, all the twenty female PD patients exhibited migration pattern B (similar to controls) with respect to the two reactions of exon 3 of FOXA1 gene.

### 3.6. Association Analysis

Table 5 shows the mean values showing the effect of SSCP patterns on NURR1 and FOXA1; SSCP analysis of the amplicon including exon 4 revealed that on average patterns B and D significantly (*p* < 0.005) influenced the NURR1 gene expression in male PD. SSCP band patterns B and A at FOXA1 exon 3 significantly (*p* < 0.005) influenced the FOXA1 gene expression in male PD. To our knowledge, this is the first report of male PD patients with gene variation in exon 4 of NURR1 and exon 3 of FOXA1. However, no genetic variation was found in exon 4 of NURR1 and exon 3 of FOXA1 in female PD patients. Furthermore, sex dependent genetic variation was demonstrated in exon 4 of NURR1 and exon 3 of FOXA1 of healthy controls, respectively.

## 4. Conclusions

In conclusion, these findings endorse the fact that common haplotype variation in transcription factors might have important and clinically relevant associations with PD. We anticipate that these findings will have implications for our understanding of gender differences in PD and also more broadly with potential applications in risk stratification, prognostication, and the development of appropriately targeted-treatment strategies. However, because only a small number of samples and only exon 4/3 region were investigated, a much larger association study is warranted in further studies.

## Figures and Tables

**Table 1:** Genes with corresponding primers which are used for gene expression study.

Gene	Primers sequence	Size (bp)	Accession no.
NURR1	Forward primer 5′ CGGGTCGGTTTACTACAAG 3′	111	AB017586.1
	Reverse primer 5′ TGGTGGAAGTTGTGGAGAG 3′		

FOXA1	Forward primer 5′ GTTACAGGGAGGACTACCA 3′	111	NM_004496.3
	Reverse primer 5′ TCCAAGGCAGTTCCAATAC 3′		

ACTB	Forward primer 5′ TCGTGCGTACATTAAGG 3′	175	X00351.1
	Reverse primer 5′ AAGGAAGGCTGGAAGAGT 3′		

**Table 2:** Genes with corresponding primers which are used for SSCP analysis.

Gene	Primers sequence		Size (bp)
NURR1	Exon 4 (1)	Forward 5′ AGACACGGGCTCAAGGAACC 3′	349
		Reverse 5′ ACTGCTCACACGGCTATCTCTG 3′	
	Exon 4 (2)	Forward 5′ CCATTTCTGTAACCCTCCTAGC 3′	416
		Reverse 5′ CCACCCACGCAACATTTAGT 3′	

FOXA1	Exon 3 (1)	Forward 5′ TCGGAGCAGCAGCATAAG 3′	488
		Reverse 5′ GGCAAGGAAGGAGGAGAAT 3′	
	Exon 3 (2)	Forward 5′ GAGAACGGCTGCTACTTG 3′	300
		Reverse 5′ GGAGGCTGGAGTCTTCAA 3′	

**Table 3:** NURR1 gene expression versus *β*-actin as internal control in all groups.

Group	Gender	Age	Number of samples used	NURR1 mRNA mean ± SME	*p *value
HC	Male	68.45 ± 1.282	20	1.208 ± 0.36	<0.05
PD	Male	68.85 ± 1.19	20	0.5084 ± 0.35	

HC	Female	65.8 ± 1.133	10	1.13 ± 0.076	<0.05
PD	Female	65.7 ± 1.202	10	0.803 ± 0.028	

**Table 4:** FOXA 1 gene expression versus *β*-actin as internal control in all groups.

Group	Gender	Age	Number of samples used	FOXA1 mRNA mean ± SME	*p *value
HC	Male	68.45 ± 1.282	20	1.373 ± 0.77	<0.05
PD	Male	65.85 ± 1.19	20	0.4884 ± 0.045	

HC	Female	65.8 ± 1.133	10	1.681 ± 0.201	<0.05
PD	Female	65.7 ± 1.202	10	0.7436 ± 0.07	

**Table 5:** Mean values (±SEM) of mRNA expression (2^−Δct^) with PCR-SSCP patterns of NURR1 and FOXA1 gene.

Gene	Gender	Condition	2^−ΔCT^	SSCP patterns		Significance
				PCR1	PCR2	
NURR1	Male	HC	1.208	A	B	<0.05
		PD	0.508	D	B (0.2)	
					D (0.8)	
	Female	HC	1.13	B	B	NS
		PD	0.80	B	B	

FOXA1	Male	HC	1.327	C	B	<0.05
		PD	0.4884	B	A	
	Female	HC	1.681	D	D	NS
		PD	0.7436	D	D	

*Note*. NS represents “not significant” and B (0.2) and D (0.8) show frequency of the patterns B and D

PCR1: PCR amplification of exons in reaction 1; PCR2: PCR amplification of exons in reaction 2.
